# Polycystic ovary syndrome and its multidimensional impacts on women’s mental health: A narrative review

**DOI:** 10.1097/MD.0000000000038647

**Published:** 2024-06-21

**Authors:** Haya Almhmoud, Lara Alatassi, Mouna Baddoura, Joudy Sandouk, Mohamad Zafer Alkayali, Hasan Najjar, Basem Zaino

**Affiliations:** a Faculty of Medicine-Tishreen University, Lattakia, Syria; b Department of Laboratory Medicine, Aleppo University Hospital, Aleppo, Syria; c Faculty of Medicine-Damascus University, Damascus, Syria; d Faculty of Medicine -Aleppo University, Aleppo, Syria; e Department of Pathology, Tishreen University Hospital, Lattakia, Syria.

**Keywords:** anxiety, cognitive-behavioral therapy, depression, lifestyle interventions, mental health, polycystic ovary syndrome, quality of life

## Abstract

Polycystic ovary syndrome (PCOS) is a common endocrine disorder that affects approximately 8% to 13% of women of reproductive age worldwide. It is characterized by a range of symptoms, including hirsutism, acne, and menstrual irregularities, and poses a significant psychological burden, including anxiety and depression. The evolving definition of PCOS, from the 1990 NIH conference to the 2003 Rotterdam conference, highlights its multifaceted nature, encompassing metabolic, reproductive, and psychological aspects. This overview aims to elucidate the complex interplay between PCOS’s physiological and psychological dimensions. It focuses on understanding the heightened risk of psychiatric disorders, including depression and anxiety, among women with PCOS and explores the contributing factors, such as obesity, body image issues, and stress. The etiology of PCOS involves a complex mixture of genetic, hormonal, and lifestyle factors that contribute to its pathophysiology and the associated mental health challenges. Stress, in various forms, including metabolic, inflammatory, oxidative, and emotional, is identified as a significant contributor to the pathogenesis of PCOS. Management strategies highlighted include lifestyle modifications, dietary and exercise interventions, and psychological therapies, underscoring the need for comprehensive and integrated care approaches that address the broad spectrum of PCOS effects. A multifaceted treatment approach that goes beyond just the physical symptoms of PCOS to also include its significant psychological effects is emphasized, reinforcing the necessity for a comprehensive, integrated care strategy to manage this complex condition effectively.

## 1. Introduction

Polycystic Ovary Syndrome (PCOS) is a prevalent endocrine disorder, affecting approximately 8-13% of women of reproductive age worldwide.^[1]^ A complex interplay of factors, including ethnicity, environmental influences, genetics, and diagnostic criteria, influences the incidence of PCOS. Women diagnosed with PCOS frequently exhibit symptoms such as hirsutism, affecting about 70% of those diagnosed, alongside acne, menstrual irregularities, and obesity.^[2,3]^

Moreover, PCOS is associated with heightened risks of several serious health conditions, including diabetes mellitus, hypertension, lipid disorders, and metabolic syndrome, highlighting the critical need for comprehensive management strategies.^[4,5]^

The impact of PCOS extends beyond physical symptoms as there is a notable psychological impact; women with PCOS are at higher risk of mental health issues, including depression, anxiety, bipolar disorder, obsessive-compulsive disorder, somatization, eating disorders, and reduced sexual satisfaction.^[6,7]^ Anxiety and depression are particularly prevalent among women with PCOS, with a reported 64.1% experiencing depressive disorders.^[8,9]^ The disorder can significantly diminish the overall quality of life, adversely affected by weight gain, hirsutism, infertility, and menstrual irregularities.^[9,10]^ Moreover, Living with PCOS can challenge societal norms regarding appearance, leading to social withdrawal and negatively impacting sexual relationships.^[11]^ This encourages mentioning cultural perspectives that influence the rate of psychological response to PCOS, affecting how symptoms are perceived and managed.^[12]^ Interestingly, studies indicate that these challenges in sexual self-esteem are not directly correlated with androgen levels.^[7]^

In the mid-20th century, treatments for PCOS were primarily concerned with fertility, utilizing hormone supplements and methods like artificial insemination.^[13]^ However, as understanding of the disorder has evolved, so too have treatment methodologies beyond fertility, and by the turn of the millennium, there was a growing recognition of the importance of managing weight and increasing physical activity as essential components of PCOS care.^[14,15]^ Regular exercise was found to not only assist with weight management but also to significantly improve mental health, leading to a better quality of life for those affected by PCOS.^[15,16]^

Dietary management has also been recognized as a crucial component of PCOS care. High-protein, low-carbohydrate diets, for example, have been noted for their positive impact on metabolic symptoms and mental health outcomes.^[17,18]^ Additionally, Cognitive Behavioral Therapy (CBT) has become a cornerstone in the management of PCOS, offering significant benefits in reducing depression and anxiety, thus enhancing overall quality of life.^[19,20]^

It equips women with adequate skills to manage their thoughts and emotions while aiding in weight loss and improving stress responses.^[20]^ This makes it an integral and useful tool in PCOS management and broader mental health treatment.

**Figure 1.**
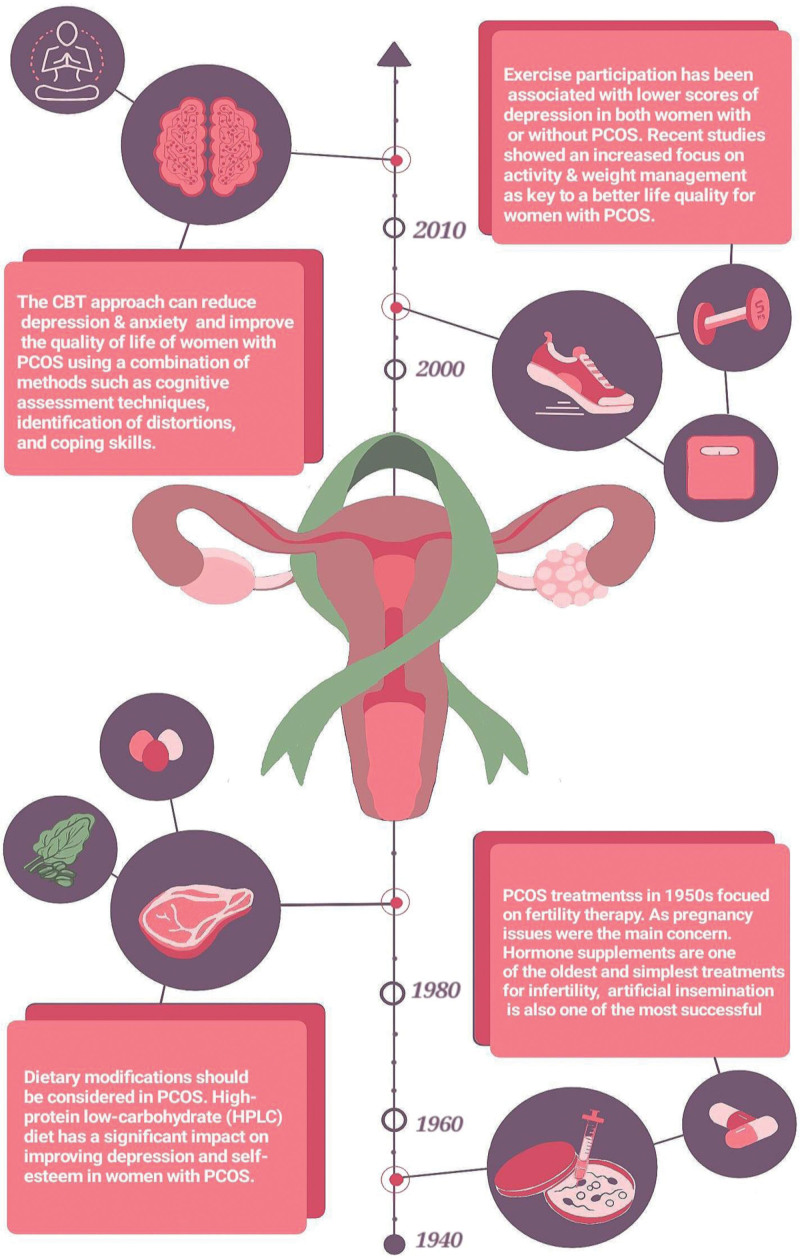
Evolution in understanding and treating mental health in PCOS. PCOS = polycystic ovary syndrome.

The holistic approach to treating PCOS reflects a broader movement in healthcare that values the interrelation between physical and mental health and promotes all-encompassing treatment strategies that focus on the patient’s overall wellness. We have illustrated the historical progression of treatment strategies for PCOS in Figure 1.

This review aims to explore the intricate relationship between mental health and PCOS, emphasizing the urgent need for advanced methods to assess and treat mental health issues among PCOS patients. Through a comprehensive examination of current research and treatment approaches, we seek to illuminate the complexities of PCOS management and advocate for a more integrated approach to care.

## 2. PCOS – definitions

PCOS is the most prevalent endocrinopathy affecting women in their reproductive years. This complex condition encompasses aspects of metabolism, reproduction, and psychology. Its prevalence varies depending on the definitions and population investigated.^[21]^

The journey to establish diagnostic criteria for PCOS began at a pivotal 1990 NIH conference, where the proposed diagnostic criteria for PCOS included oligo-anovulation, hyperandrogenism (HA), and/or hyperandrogenemia, coupled with the exclusion of related diseases.^[22]^

In 2003, the Rotterdam Conference expanded these criteria by introducing polycystic ovary morphology to the previous diagnostic criteria. This addition mandated that PCOS diagnosis requires two of the 3 following criteria: oligo or anovulation, clinical or biochemical HA, and polycystic ovary morphology on ultrasound, after ruling out other disorders.^[23]^

Further refinement came in 2013 when the Endocrine Society Practice Guideline suggested utilizing adult criteria, which included chronic irregular menstrual periods and HA, to diagnose PCOS in teenage girls.^[24]^

The Evidence-Based International Guidelines endorsed the Rotterdam criteria, which assembled healthcare providers to discuss diagnostic criteria and provide beneficial treatment choices.^[21]^

The guidelines recommended following multiple diagnostic criteria like monitoring irregular cycles and ovarian morphology, hormonal testing, and screening for depression in all adults and adolescents with PCOS, using regionally validated screening tools.

Recent updates have introduced the measurement of anti-Müllerian hormone levels as an alternative to ultrasound for adults and underlined the necessity of spotting broader features of PCOS, such as metabolic risk factors, in the diagnostic process.^[21]^

While many theories have been proposed to clarify the underlying causes and mechanisms of PCOS, the exact causes are not fully understood.^[25]^ Numerous factors play a role in the pathophysiology of developing PCOS: hormonal imbalance, insulin resistance, genetics, and epigenetics.^[26]^ Past studies support the hypothesis of prenatal androgen in PCOS pathophysiology, while other studies show there is no link between maternal androgen and the development of PCOS in youth.^[27–30]^

## 3. Etiopathogenesis of PCOS: physiological and psychological insights

The impact of PCOS extends beyond physiological symptoms, significantly affecting the mental well-being of diagnosed women.^[31,32]^ Symptoms such as anovulation (lack of ovulation) and oligo-ovulation (infrequent ovulation) not only contribute to infertility but are closely linked to increased risks of psychological conditions like depression and anxiety.^[33]^

The unsuccessful treatment of infertility could exacerbate the development of these psychological challenges, leading to marital distress.^[34]^ Although the relationship between these factors remains controversial, depression, anxiety, and impaired quality of life are more prevalent in infertile women compared with fertile.^[35,36]^

The association between mental health and PCOS is multifaceted, with both non-modifiable and modifiable risk factors contributing to the increased risk of emotional discomfort, anxiety, depression, and stress in individuals with PCOS. Non-modifiable risk factors include childlessness, infertility, ethnic predisposition affecting insulin resistance, and genetic tendencies toward developing PCOS or mental disorders.^[37,38]^ On the other hand, modifiable risk factors encompass the symptoms of PCOS, including obesity, excessive body hair, acne, and infertility, which can cause emotional distress, reduction in quality of life, self-esteem, marital and social status, and mood disturbances.^[7,39–42]^ Addressing symptoms through targeted therapies may offer relief and improve quality of life.^[43]^

Insulin resistance, a contributor to hyperandrogenism and obesity, exacerbates hormonal imbalances, inflammation, and increased visceral fat. Plus, elevated cortisol levels and body mass index (BMI) are directly linked to various psychological disorders.^[44]^

Moreover, the relationship between high androgen levels and sexual function suggests that the adverse effects of androgens in females, such as acne vulgaris and hirsutism, can lead to aesthetic problems, low self-esteem, and body image disturbances, ultimately impacting the psychosexuality of patients.^[41,45]^

### 3.1. The overlooked role of stress in PCOS

**Figure 2.**
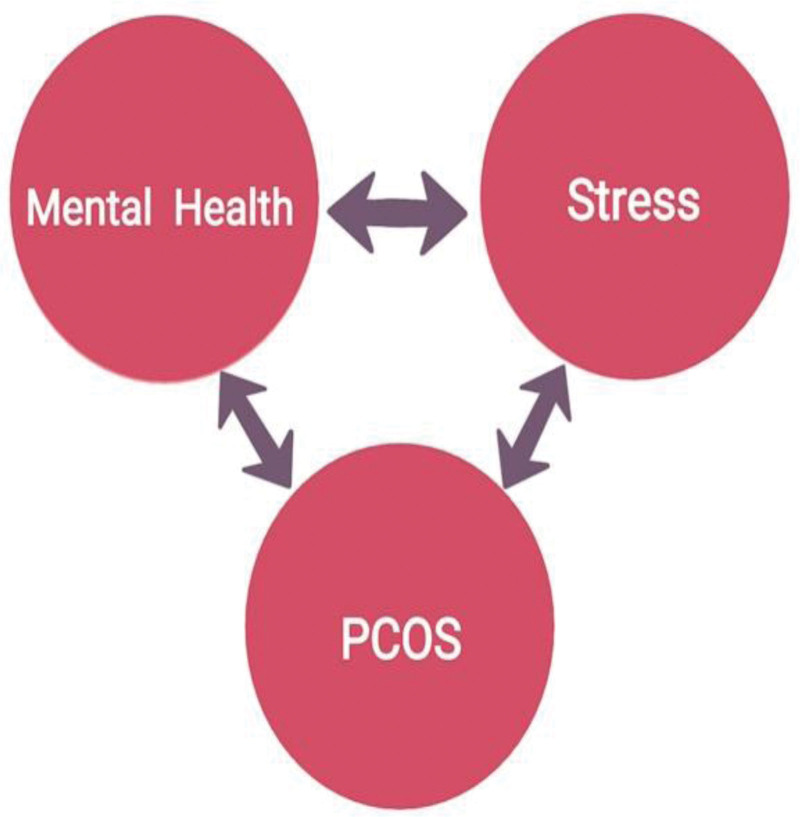
The interplay between PCOS, stress, and mental health. PCOS = polycystic ovary syndrome.

Stress significantly influences the development and exacerbation of PCOS, manifesting in various forms such as metabolic, inflammatory, oxidative, and emotional stress.^[46]^ (Fig. 2)

#### 3.1.1. Metabolic stress

Metabolic stress is the root cause of severe long-term health problems. It is the pathophysiological core of PCOS; it increases the syndrome’s psychological, metabolic, and reproductive abnormalities and creates a never-ending cycle of chronic illness.^[46]^

The body as a whole experiences critical metabolic stress that targets various organs and tissues, including: the pancreas (leads to hyperinsulinemia and insulin resistance), the liver (increased hepatic gluconeogenesis), adipose tissue (dyslipidemia), the ovaries (ovulation irregularities), and the adrenal glands (androgen excess).^[46]^

#### 3.1.2. Oxidative stress

Oxidative stress has been strongly linked to the development of metabolic issues associated with PCOS, such as obesity, insulin resistance, and endothelial dysfunction, which put PCOS-affected women at higher risk for cardiovascular disease.^[46]^

Arising from an imbalance between free radicals and antioxidants, oxidative stress can occur nearly anywhere in our biological systems; also, environmental and genetic factors play a role in contributing to oxidative stress.^[47]^ Women with PCOS have high levels of oxidative stress in both their serum and follicular fluid, which negatively affects folliculogenesis and fertility.^[48]^

The correlation between oxidative stress and psychological well-being suggests that oxidative stress does not merely affect physiological health but also contributes to the emotional distress experienced by many women with PCOS.

#### 3.1.3. Emotional stress

Unfortunately, neither PCOS patients nor general practitioners view psychological distress as a prominent feature of the disease, potentially indicating a lack of awareness about emotional distress.^[49]^ It appears that women who receive a late diagnosis may face stress and a threat to their quality of life. So, when PCOS is diagnosed later in life, patients feel upset, and the psychological pressure on women increases when they receive inadequate care.^[49,50]^ Furthermore, women with PCOS experience higher levels of stress during infertility management procedures like assisted reproductive techniques compared to those without the condition. Many also display alexithymia- a personality construct characterized by an inability to regulate normal affect- indicating a profound emotional toll.^[51]^

Acknowledging the patient’s psychological state as a primary concern is pivotal in the management of PCOS. Therefore, an effective treatment regimen must encompass a combination of pharmacological interventions and meditation.^[52,53]^

## 4. Types and manifestations

Mental health issues in the context of PCOS manifest in a wide set of psychological conditions, including depression, anxiety, eating disorders, bipolar disorder, and more.

Depression has a significant correlation with body image issues in women with PCOS, a situation that has been exacerbated by the rising incidence of body image distress among this group in recent times.^[54,55]^

According to studies, higher levels of anxiety are related to single relationship status and unemployment, which could further escalate mental health issues in PCOS women.^[56]^ Studies reveal that individuals with PCOS are nearly 3 times more likely to experience depressive symptoms and face a quadruple increase in anxiety risk.^[6]^

Beyond depression and anxiety, PCOS is linked with an increased risk of several psychiatric disorders, including bipolar disorder, obsessive-compulsive disorder, and somatization.^[6]^ Interestingly, the long-term use of valproate, a medication used in the treatment of bipolar disorder, has been associated with a higher risk of developing PCOS, indicating potential bidirectional relationships between PCOS and certain psychiatric conditions.^[57,58]^

Obesity, commonly seen in PCOS, not only exacerbates depression but also, when addressed, may alleviate symptoms.^[59]^

Eating disorders, which primarily affect young women, are also associated with PCOS. These include anorexia nervosa, bulimia nervosa, and atypical eating disorders.^[60]^ Women with PCOS have a higher risk of experiencing eating disorders compared to non-PCOS women.^[61]^

Interestingly, mental health concerns can arise from PCOS symptoms itself, for example skin manifestations such as acne and hirsutism are strongly associated with depression and severe anxiety.^[7,62]^

## 5. Screening tools overview

Challenges like diminished self-confidence, introversion, and fear of social judgment complicate the identification of mental health issues in women with PCOS.^[63]^ The diverse range of symptoms and severity levels in PCOS makes it harder to diagnose early. This leads to treatments focusing on symptoms instead of using well-researched plans to address the issue.

Not only the symptoms but also the evaluation methods employed to assess psychiatric disorders in women with PCOS displayed considerable diversity. PCOS is recognized as a syndrome that affects multiple systems within the body, thus, patients often seek consultations with different specialists such as dermatologists, gynecologists, and psychologists, thus, accurate diagnosis of PCOS requires well-coordinated healthcare across those specialties.^[64]^

Several assessment tools have been developed over the years; those are to assess the psychological status of a patient with PCOS (Table 1).

**Table 1:** Scales used for assessing psychological health in women with PCOS.

	Toronto Alexithymia Scale (TAS-20)	Body Uneasiness Test (BUT)	Minnesota Multiphasic Personality Inventory-2 (MMPI-2)	Short-Form Health Survey (SF-36)	Difficulties in Emotion Regulation Scale (DERS)
Measured items	20-item self-reported alexithymia scale	71 self-reported items questionnaire divided into 2 parts: BUT-A scale of items examining body form, weight dissatisfaction, avoidance, obsessive control activities, and emotions of separation and dissatisfaction of one’s own body. BUT-B is a 37-item scale that assesses specific concerns about certain bodily parts, shapes, or functions, such as buttock odor and flushing.	The following scales of 567 questions – true or false – are intended to give psychopathological information in 3 validity scales, 10 clinical scales, sixteen supplemental scales, and fifteen content scales	Multi-item scale that evaluates 8 health concepts: 1) physical activity limitations due to health issues 2) social activity limitations due to physical or emotional issues 3) physical activity limitations due to health issues in usual role activities 4) the sensation of pain 5) general mental health (psychological distress and well-being) 6) usual role activities limitations due to emotional problems 7) vitality (strength and exhaustion) 8) general health perception	A 36-item self-report questionnaire covering 6 aspects of emotion regulation.
Score calculation criteria	A score of 61 or more indicates alexithymia, whereas scores between 51 and 60 indicate borderline alexithymia.		A *t* value of 65 indicates psychological dysfunction.	Each scale is directly transformed into a 0–100 scale on the assumption that each question carries equal weight. The lower the score, the more disability. The higher the score, the less disability, i.e., a score of zero is equivalent to maximum disability, and a score of 100 is equal to no disability.	A scale of 1 means “rarely [0–10%]” and 5 means “almost always [91–100%].” Higher scores indicate more significant challenges with emotion management.

The data for TAS-20, BUT, and MMPI-2 scales are from Scaruffi E, Franzoi IG, Civilotti C, et al. Body image, personality profiles and alexithymia in patients with polycystic ovary syndrome (PCOS). J Psychosom Obstet Gynaecol. 2019;40:294–303. doi:10.1080/0167482X.2018.1530210

The data for the SF-36 scale are from Ware JE, Jr, Sherbourne CD. The MOS 36-item short-form health survey (SF-36). I. Conceptual framework and item selection. Med Care. 1992;30:473–83. and Health-Related Quality-of-Life Tools – Immune Disorders. https://doi.org/10.1016/B978-0-12-804217-5.00023-0

The data for the DERS scale are from Hallion LS, Steinman SA, Tolin DF, Diefenbach GJ. Psychometric properties of the difficulties in emotion regulation scale (DERS) and its short forms in adults with emotional disorders. Front Psychol. 2018;9:539. https://doi.org/10.3389/fpsyg.2018.00539

PCOS = polycystic ovary syndrome.

The Body Uneasiness Test (BUT) is a 71-item self-administered survey used to examine body image problems and related psychopathologies in therapeutic settings. The Minnesota Multiphasic Personality Inventory-2 (MMPI-2) is a standardized psycho-diagnostic instrument composed of 567 binary (true or false) questions that offer psychopathological information on various scales: 3 validity scales, 10 clinical scales, sixteen supplemental scales, and fifteen content scales.^[65]^

However, the most commonly used tools for evaluating the quality of life in women with PCOS are the SF-36 (Short Form Health Survey) and PCOSQ (Polycystic Ovary Syndrome Questionnaire). These instruments have effectively identified areas for improvement in quality of life and capturing various aspects.^[66]^

The PCOSQ consists of 26 items measuring 5 aspects of Health-Related Quality of Life: emotions, body hair, weight, infertility problems, and menstrual problems. Each question on the PCOSQ corresponds to a 7-point scale, allowing for comparison with the SF-36.^[67]^ General instruments like the SF-36 have revealed that women with PCOS have lower Health-Related Quality of Life compared to women without the condition.^[7]^

Women with PCOS have been found to score higher on the SCL-90-R’s aggression category, indicating a higher prevalence of obsessive-compulsive signs and symptoms compared to women without PCOS.^[7]^ It is recommended that mental health symptomatology, especially depression, should be included in the initial screening process for women with PCOS, and a reliable method for diagnosing depression should be implemented in gynecology and obstetrics primary care clinics.^[7]^

Checking for irregular eating habits is advised as well, because undiagnosed eating disorders such as anorexia nervosa, bulimia nervosa, and binge eating may worsen the physical health of PCOS-affected women.^[7,66]^

Physicians have the potential to raise awareness, aid in prompt diagnosis, and help patients manage complications of PCOS through digital tools and support groups. A consensus on PCOS counseling is necessary due to the lack of clear standards available to address frequently overlooked elements of PCOS.^[66]^

## 6. Clinical presentation of mental health disorders in PCOS

Although there has been much research on the appearance of women with PCOS, there has not been enough attention given to their psychological aspects, such as personality, how they interact with others, and the way they manage their emotions. These factors have been overlooked in research despite their importance in determining the best treatment options for this condition.^[68]^

The comorbidity of PCOS includes mood disorders (18.2–81%) and anxiety disorders (2.8–35.7%).^[69,70]^

In 2018, a study was conducted to compare psychological variables between women with Polycystic Ovary Syndrome (PCOS) and a control group without the condition. The research utilized 3 psychological assessment tools: the Toronto Alexithymia Scale TAS-20, the BUT, and the MMPI-2.^[65]^ The findings revealed that women with PCOS scored higher than those in the control group across several dimensions. Specifically, on the TAS-20, women with PCOS demonstrated greater difficulty in identifying and describing feelings, more externally oriented thinking, and higher overall alexithymia scores. Additionally, the BUT results indicated that the PCOS group had higher scores in the Positive Symptom Complete Index and the Global Severity Index scales, suggesting greater body uneasiness.^[65]^

The MMPI-2 results further highlighted significant differences, with the PCOS group showing elevated scores in 8 out of 10 clinical scales, including hypochondriasis, depression, hysteria, psychopathic deviation, paranoia, psychasthenia, schizophrenia, and social introversion. Moreover, they scored higher in several content scales related to anxiety, fears, obsessiveness, depression, health concerns, low self-esteem, social discomfort, family problems, work interference, and negative treatment indicators. Supplementary scales also showed higher scores in the PCOS group for posttraumatic stress disorder, and marital distress.^[65]^

Additionally, women with PCOS often struggle with body image issues, perceiving their bodies as inadequate or flawed. They also wrestle with deep-seated negative perceptions and feelings toward their bodies.^[65]^ Another meta-analysis aimed to determine the prevalence, mean level, standardized mean difference and probability of depression based on the research conducted with the Hospital Anxiety and Depression Scale. The overall likelihood of depression in PCOS patients was more than 2.5-fold higher than in healthy women^[71]^

Additionally, a systematic review and meta-analysis were published comparing women with PCOS to control groups on anxiety and depression. Results found that women with PCOS on average tend to experience mildly elevated anxiety and depression, significantly more than women without PCOS. Women with PCOS with lower BMI tended to have slightly lower anxiety and depression scores, suggesting that having a lower BMI reduces anxiety and depression.^[72]^

In a cross-sectional study, 418 women with and without PCOS completed assessments on emotion dysregulation, rumination, non-suicidal self-injury, suicidal ideation, and Difficulties in Emotion Regulation Scale. Results showed that women with PCOS reported significantly higher levels of all variables compared to those without PCOS.^[73]^

Furthermore, a case-control study involving 240 infertile females utilized questionnaires such as the fertility problem inventory, female sexual function index, Beck depression inventory-II, and Toronto alexithymia scale (TAS-20) to gather data. The results indicated that women with polycystic ovary syndrome (PCOS) exhibited significantly elevated levels across all variables compared to those without PCOS. Specifically, infertile females with PCOS reported higher levels of infertility stress and difficulties in recognizing and articulating their emotions when compared to those without PCOS.^[51]^

When interpreting the data of the abovementioned studies, it is essential to weigh the evidence accordingly. Small sample sizes, lack of randomized controlled trials, and potential biases can affect the reliability and generalizability of these study conclusions. We emphasize the need for more rigorous research designs in future studies to strengthen the evidence base regarding mental health disorders in women with PCOS.

## 7. Preventive therapy

Preventing the deterioration of mental health is pivotal in the management of PCOS. It empowers women to adopt a healthier lifestyle, potentially preventing the progression of symptoms and subsequent mental health issues.^[74]^

The emotional impact of coping with PCOS, including concerns about infertility, body image, and self-worth, can contribute to poorer mental health outcomes. Therefore, optimizing physical activity as a preventive treatment for PCOS should take into account the mental health status of affected women and its potential interactions with physical activity. The first Evidence-based Guideline recommends this approach for assessing and managing PCOS.^[74,75]^

Lifestyle changes and weight management are crucial for the treatment of PCOS.^[76]^ Including behavioral and psychological strategies such as goal setting, self-monitoring, cognitive restructuring, problem-solving, and relapse prevention can improve the outcomes of weight management programs for women with PCOS. Strategies that target improved motivation, social support, and psychological well-being are also essential and can be tailored to women at different reproductive life stages.^[77]^ It is important to note that the relationship between PCOS and behavioral and psychological therapies is still young in research. However, some recognizable efforts in studying the associations between PCOS and the following preventive measures have been made.

### 7.1. Physical activity

Physical activity is a critical component of primary PCOS management, as it is an effective therapeutic option for the reproductive and metabolic features of the condition.^[74,75]^ Promising evidence supports vigorous aerobic exercise in improving body composition, cardiorespiratory fitness, and insulin resistance.^[78]^ Preliminary data suggests that physically inactive women with PCOS have higher depression scores compared to those who are physically active.^[74]^

### 7.2. High-intensity intermittent training (HIIT)

Clinical trials in PCOS showed that HIIT prescribed for 12 to 24 weeks can effectively improve important clinical outcomes, including insulin sensitivity, body fat percentage, LDL-cholesterol, C-reactive protein, and psychological outcomes. Such studies have posted clinical practice guidelines, which recommend that women with PCOS engage in ≥ 90 minutes of HIIT training weekly.^[79]^

### 7.3. Diet

It is essential to recognize that many individuals with PCOS experience hormonal imbalances, high cholesterol levels, and obesity. Therefore, simply engaging in physical activity may not be sufficient for weight loss.

A balanced and monitored diet is crucial for managing weight, improving self-esteem, and reducing metabolic stress, which can have long-term health implications. This stress can exacerbate the reproductive, metabolic, and psychological issues associated with PCOS, creating a cycle of chronic illness.^[46]^

A small study of women with PCOS assigned to a ketogenic, low-carbohydrate diet for 6 months reported significant improvement in their weight and fertility.^[80]^ Also, diets with a low glycemic load may influence appetite-regulating hormones including increasing glucagon and reducing ghrelin in women with PCOS. This diet is especially relevant since altered satiety hormones in women with polycystic ovarian syndrome may contribute to obesity.^[81]^

Additionally, the pulse-based diet, consisting of split-peas, dry beans, lentils, and chickpeas, has been associated with positive metabolic effects such as lowering postprandial blood glucose and insulin concentrations, and decreasing hypercholesterolemia, blood pressure, and obesity in women with PCOS.^[82]^ A case-control study was performed on 225 patients newly diagnosed with PCOS and 345 healthy women, and analyzed 3 major dietary patterns including Western, plant-based, and mixed. The study found that Western and plant-based dietary patterns were associated with an increased risk of PCOS, while moderate adherence to the mixed dietary pattern was associated with a reduced risk of PCOS.^[83]^

While some studies have shown positive outcomes with different diets, there is limited evidence to recommend a specific dietary composition for PCOS.^[84]^

### 7.4. Sleep

In addition to diet and exercise, addressing psychological well-being and sleep patterns is essential for individuals with PCOS. Many women with PCOS experience emotional distress and sleep disturbances, which can hinder their ability to make positive lifestyle changes.

Women with PCOS were recruited through social media for a cross-sectional study conducted during the COVID-19 lockdown. The study utilized online surveys that included demographic information, COVID-19-related questions, and validated questionnaires such as the Insomnia Severity Index. The results revealed a negative impact on sleep among participants, which was found to be associated with reduced quality of life and higher levels of depression and stress.^[85]^ However, research on clinical interventions to improve these areas is currently lacking.^[78,86]^

### 7.5. Early intervention

Early intervention is critical for preventing long-term complications and improving fertility in women with PCOS. Women who receive treatment later in life are more likely to be obese, insulin-resistant, and less likely to have undergone surgical interventions due to advanced complications. Starting treatment early can also help address future mental health issues that may arise from the progression of PCOS.^[87,88]^ Psychological interventions such as relaxation techniques, logotherapy, medication, and electroconvulsive therapy have been shown to help treat anxiety and depression in individuals with PCOS.^[88]^

Designing individualized programs that incorporate physical activity, monitored diet, proper medication, and therapy for young women with PCOS can lead to improved mental health and quality of life. Early implementation of these programs can also reduce long-term medical and treatment costs for these patients.^[75,89]^

## 8. Management

### 8.1. Psychological therapies and pharmacotherapy

When it comes to PCOS management, an interdisciplinary approach is crucial to treat the full symptomatic spectrum of this condition. While women are treated medically, the successful use of psychotherapy made it the first line of treatment for PCOS-related mental health issues.^[90]^

CBT is a form of psychotherapy that is the most common treatment for anxiety and depression, making it highly accessible for women with PCOS. CBT helps patients reconstruct their behaviors, build new thought patterns, and ultimately reframe healthier mindsets for a better quality of life.^[20]^ In a clinical analysis study, CBT notably improved the mean score of quality of life in menstrual problems, weight, infertility, and emotional problems. The sessions focused on practicing cognitive skills such as identifying automatic thoughts and cognitive distortions. The participants in the counseling group reported a significant decrease in weight; the quality-of-life score was increased compared to the lifestyle change group. Some meetings included self-concept, breathing techniques and timing, nutrition, muscle relaxation, stress management, and positive expression.^[88,91]^

**Figure 3.**
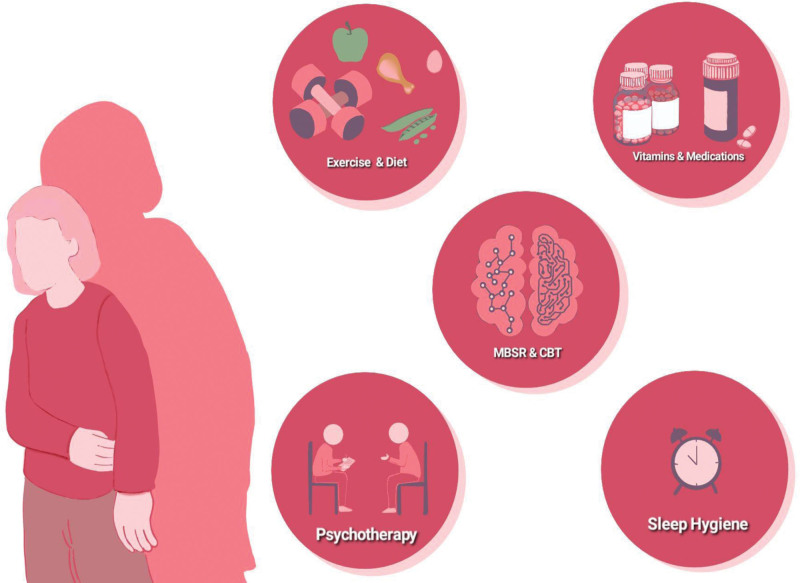
Illustration representing various management guidelines.

The pharmacotherapeutic treatment in PCOS is primarily symptomatic and may involve lifestyle interventions combined with medications such as metformin, oral contraceptives, and antiandrogens.^[92]^ Studies have shown how lifestyle changes, exercise, diet, and pharmacotherapies impact PCOS’s physical, clinical, and biochemical aspects^[93,94]^ (Fig. 3).

The use of oral contraceptive pills (OCP) to reduce psychological distress is still under speculation, but numerous studies reported a significant reduction in depressive symptoms. The mechanism is believed to be associated with lower androgen levels, improved menstruation, and PCOS symptom relief like hirsutism. However, there are still very few studies on the exact link between OCP use on mental health and depression.^[18]^ Additionally, a clinical case study involving a PCOS patient with no prior psychiatric history who presented with suicidal ideation suggested a potential role for bupropion and naltrexone in managing PCOS symptoms, depression with suicidal ideation, and trichotillomania. Bupropion increases centrally available dopamine and norepinephrine levels by blocking their reuptake.^[95,96]^

While medical treatment is a crucial aspect of managing PCOS, the psychosocial component is often overlooked. Therefore, counseling is pivotal in PCOS management, improving clinical outcomes and patient satisfaction. The adverse biopsychosocial effects highlight the importance of raising awareness and addressing these conditions promptly and effectively. It is essential to recognize that solely relying on biomedical approaches may not yield optimal results and that psychosocial and social support is necessary for comprehensive care. The core pillars of detecting, preventing, and treating the physiological and psychological issues associated with PCOS are screening, assessment, and counseling. These are pivotal in addressing the comprehensive needs of individuals with PCOS.^[66]^ Patient counseling involves providing advice and education to patients and their families about medication and lifestyle changes and addressing psychological factors to improve their Quality of Life. Counseling interventions can help release emotional distress, establish positive relationship patterns, and support healthy behaviors, enhancing patient care.^[97,98]^

Digital forms of psychotherapy, such as acceptance and commitment therapy, behavioral activation, interpersonal psychotherapy, mindfulness interventions, and problem-solving therapy, are effective. An Indian study demonstrated the benefits of digital therapeutics in girls with PCOS using a wellness application, which improved patient engagement and treatment adherence.^[99]^

For successful management of PCOS, pharmacological and psychological treatment approaches are both crucial and have shown multiple promising results.^[100]^

### 8.2. New and emerging treatments

Management of PCOS cardiovascular, metabolic, and reproductive symptoms had the attention of researchers throughout history. Still, with the rise in mental health awareness in the 21st century, many new attempts at finding mental health therapies for women with PCOS have been made.

Recent studies suggest that complementary and alternative medicine treatments could be helpful as an addition to conventional medical management of women with PCOS, including mindfulness.^[101]^ Mindfulness-based stress reduction could be of great benefit for overweight/obese women, including those with polycystic ovary syndrome (PCOS), as it has been shown to reduce psychological distress while enhancing quality of life in other patient populations. Preliminary studies suggest that mindfulness-based stress reduction may also benefit blood pressure and blood glucose136. Mindfulness techniques include the clinical application of non-judgmental acceptance of psychological distress, thereby lowering the tendency to contemplate the symptoms. Several studies have shown that brain concentration to “moment-to-moment” awareness of one’s present thoughts, emotions, and sensory experiences in a non-judgmental manner, in affiliation with neuroplasticity, promotes stress relief due to enhanced mental repose.^[101,102]^

Progressive resistance training (PRT) improved muscle strength, reduced pain, and total depression and anxiety scores in women with and without PCOS.^[103]^ A study randomized participants into an experimental PRT or no-exercise control group. The PRT group reported significant improvement in physical capacity, vitality, social functioning, and mental health compared to the control.^[104,105]^ A suitably powered clinical trial is required to confirm these findings and answer novel research questions about prescribing PRT as a therapeutic intervention in PCOS.

New studies have shown that insulin resistance is related to increased depression risk.^[106]^ The association between insulin resistance and depressive mood calls for further investigation to identify potential therapies. Recent research has also explored the use of newer insulin sensitizers such as inositol, Glucagon-like peptide-1 agonists, Dipeptidyl peptidase-4 inhibitors, and sodium-glucose transport protein 2 inhibitors for the management of PCOS.^[107]^ One study demonstrated a notable depletion in the homeostatic model assessment of insulin resistance 3 weeks after administering a single oral dose of 300,000 IU of vitamin D3 to 11 obese women with PCOS.^[108,109]^ Another study explored cinnamon extract (a traditional herb), as it has been shown to potentiate the insulin effect through the upregulation of glucose uptake in cultured adipocytes.^[110,111]^ The use of supplements, including omega-3 and vitamin E, due to improved insulin sensitivity and inflammation, remains the most effective treatment strategy for PCOS subjects.^[112]^ Omega-3 and vitamin E co-supplementation effectively enhance mental health parameters and gene expression of PPAR-γ, IL-8, and TNF-α in women with PCOS.^[113]^ These studies show a new perspective on treating PCOS-related mental health issues through physiological management.

Also, several studies report that PCOS is associated with a decrease in microbial diversity and composition.^[114]^ Furthermore, studies have shown that patients with psychological disorders, depression, and bipolar disorder, have significant differences in the composition of their gut microbiome.^[115]^

Probiotics/symbiotic supplementation may enhance weight loss during diet programs and additionally positively affect metabolic and inflammatory factors by improving the intestinal microbiome as well as potentially improve mood and reduce anxiety in PCOS patients.^[116]^

Furthermore, sleep disorders are prevalent in patients with PCOS. These disorders include daytime sleepiness (hypersomnia), obstructive sleep apnea, and sleep breathing disorders that can seriously impair sleep quality in these patients.^[117,118]^ Given the substantial impact of poor sleep on PCOS patients, including its association with depression, increased insulin resistance, and obesity, it is crucial to prioritize sleep hygiene, known as the set of behavioral and environmental advice that is planned for encouraging healthy sleeping and were developed to treat sleep disorders, as a cornerstone of lifestyle modifications for these individuals.

Another study demonstrated that giving melatonin supplementation for 12 weeks to PCOS women had beneficial effects on mental health status and insulin levels. This suggests that melatonin supplementation may present great therapeutic potential for women with PCOS.^[119,120]^

One newer randomized study explored technology in helping PCOS patients. This research demonstrated that a mobile health application program based on the Transtheoretical Model can lead to long-term reductions in BMI, waist circumference, anxiety, and depression, as well as improvements in exercise and diet adherence among patients with PCOS.^[121]^

New treatments for mental health issues in PCOS require continuous research to optimize the best care for these patients with such chronic diseases.

## 9. Limitations of the review

The underlying pathophysiology of PCOS may contribute to mental health disorders; however, it is unclear whether women with PCOS have a predisposition to these disorders or are more susceptible due to the pathophysiology.

Discussing mental health disorders can be challenging for patients due to the stigma surrounding mental health in many societies. This caution can limit the availability of research studying the links between PCOS and mental health disorders, making it difficult to draw definitive conclusions or identify patterns. Furthermore, there is a scarcity of studies exploring the relationship between PCOS and mental health disorders, contributing to a lack of awareness about the psychological aspects associated with PCOS. This gap in awareness may lead to a potential gap in care for these patients. Many studies on mental health in PCOS use cross-sectional designs, which only provide a snapshot of mental health at a single point in time. Longitudinal studies are needed to better understand the trajectory of mental health issues in women with PCOS.

Another limitation of this paper is its narrative review approach, which lacks a systematic search strategy. This can result in the omission of relevant studies and may introduce bias towards finding significant associations between PCOS and mental health issues. Consequently, this approach might lead to an overestimation of the true effect size and an excessive focus on depression and anxiety, potentially overlooking other psychological disorders that may also be significantly related to PCOS.

## 10. Conclusion

The findings of various studies suggest that women who have Polycystic Ovary Syndrome (PCOS) are more likely to experience mental health issues, such as anxiety, depression, and stress when compared to those who do not have PCOS. The reasons for this association are intricate, as chronic medical conditions are often linked to mental health issues that can negatively impact quality of life and increase the severity of depressive symptoms. PCOS, with its symptoms and comorbidities, can contribute to adverse mental health outcomes. Lifestyle interventions, including diet, exercise, and cognitive behavioral therapy, have demonstrated great promise in improving mental health in women with PCOS. Physical inactivity and obesity are risk factors for depression in these women, making interventions like a ketogenic or low glycemic index diet beneficial for managing weight. Cognitive behavioral therapy can help reduce depression and anxiety while enhancing quality of life by addressing cognitive distortions and teaching coping skills. However, studies have faced challenges in controlling for confounding factors, such as BMI, hyperandrogenism, hyperinsulinemia, and inflammation, which are associated with PCOS. In conclusion, a comprehensive approach to treating women with PCOS should include addressing their psychological symptoms, and long-term research is necessary to understand the full impact of the condition.

## Author contributions

**Conceptualization:** Basem Zaino.

**Investigation:** Lara Alatassi.

**Methodology:** Basem Zaino.

**Project administration:** Basem Zaino.

**Supervision:** Basem Zaino.

**Validation:** Basem Zaino.

**Visualization:** Haya Almhmoud, Basem Zaino.

**Writing – original draft:** Haya Almhmoud, Lara Alatassi, Mouna Baddoura, Joudy Sandouk, Mohamad Zafer Alkayali, Hasan Najjar, Basem Zaino.

**Writing – review & editing:** Haya Almhmoud, Lara Alatassi, Mouna Baddoura, Joudy Sandouk, Mohamad Zafer Alkayali, Basem Zaino.
